# Influence of Copper Nanoparticles on the Physical-Chemical Properties of Activated Sludge

**DOI:** 10.1371/journal.pone.0092871

**Published:** 2014-03-24

**Authors:** Hong Chen, Xiong Zheng, Yinguang Chen, Mu Li, Kun Liu, Xiang Li

**Affiliations:** State Key Laboratory of Pollution Control and Resource Reuse, School of Environmental Science and Engineering, Tongji University, Shanghai, China; Argonne National Laboratory, United States of America

## Abstract

The physical-chemical properties of activated sludge, such as flocculating ability, hydrophobicity, surface charge, settleability, dewaterability and bacteria extracellular polymer substances (EPS), play vital roles in the normal operation of wastewater treatment plants (WWTPs). The nanoparticles released from commercial products will enter WWTPs and can induce potential adverse effects on activated sludge. This paper focused on the effects of copper nanoparticles (CuNPs) on these specific physical-chemical properties of activated sludge. It was found that most of these properties were unaffected by the exposure to lower CuNPs concentration (5 ppm), but different observation were made at higher CuNPs concentrations (30 and 50 ppm). At the higher CuNPs concentrations, the sludge surface charge increased and the hydrophobicity decreased, which were attributed to more Cu^2+^ ions released from the CuNPs. The carbohydrate content of EPS was enhanced to defense the toxicity of CuNPs. The flocculating ability was found to be deteriorated due to the increased cell surface charge, the decreased hydrophobicity, and the damaged cell membrane. The worsened flocculating ability made the sludge flocs more dispersed, which further increased the toxicity of the CuNPs by increasing the availability of the CuNPs to the bacteria present in the sludge. Further investigation indicated that the phosphorus removal efficiency decreased at higher CuNPs concentrations, which was consistent with the deteriorated physical-chemical properties of activated sludge. It seems that the physical-chemical properties can be used as an indicator for determining CuNPs toxicity to the bacteria in activated sludge. This work is important because bacteria toxicity effects to the activated sludge caused by nanoparticles may lead to the deteriorated treatment efficiency of wastewater treatment, and it is therefore necessary to find an easy way to indicate this toxicity.

## Introduction

It is well-known that the activated sludge process is the most commonly used method for biological wastewater treatment in wastewater treatment plants (WWTPs). Pollutants such as organic matter, heavy metals, nitrogen and phosphorus can be removed via absorption or degradation by the microbes in the activated sludge system using different technologies. The enhanced biological phosphorus removal (EBPR) is one of the most used methods to remove phosphorus to keep water bodies from eutrophication [1]. It is characterized by circulation of activated sludge between anaerobic and aerobic conditions. In general, microorganisms responsible for EBPR release soluble orthophosphorus (SOP) under anaerobic conditions and then take up SOP from bulk liquid to build up intracellular polyphosphate under aerobic stages. The net phosphorus removal can be achieved by discharging excess sludge with high phosphorus content because the aerobic phosphorus uptake is greater than the anaerobic phosphorus release.

The maintenance of stable physical-chemical properties of activated sludge has reported to be important for ensuring the normal operation of WWTPs [2]. Activated sludge usually exists as flocs, which are the aggregates of suspended solids containing different groups of microorganisms and organic/inorganic particles [3]. The flocculating ability, hydrophobicity, surface charge, settleability, dewaterability and bacteria extracellular polymer substances (EPS) are the main physical-chemical properties of activated sludge that are regularly monitored [4], [5]. These properties are important for multiple reasons: The hydrophobicity and surface charge are vital for forming the activated sludge flocs and keeping their stability [6], [7]. Good flocculating ability can separate the particles from the treated wastewater and clarify the effluent turbidity. The EPS can protect microbial cells against the harsh external environmental conditions, such as the exposure of heavy metals or chemicals [8], [9]. It has also been reported that the bioflocculation, settling and dewatering capacities of activated sludge are affected by change in the EPS [10]. The operation condition and wastewater composition have been observed in the literature to affect sludge characteristics, leading to a wide variation in physical-chemical properties [11], [12]. Because of the considerable variations among wastewaters, it is important to seek general indicators that may be used for daily monitoring.

Recently, nanoparticles (NPs) with at least one dimension less than 100 nm have been applied increasingly in various fields including personal care, commercial, pharmaceutics, and military [13]. Their adverse impacts on the human and environment have attracted many concerns due to their release, and many researchers have been conducted to investigate their potential toxicity effects. The released NPs would enter into WWTPs via civil sewage systems. Studies regarding the removal of NPs and the existing species of NPs in WWTPs have been conducted recently [14]–[16]. Also, in recent literature, the influences of NPs on activated sludge [17]–[19], the change of bacterial community structure [20], [21], the changes of chemical oxygen demand (COD), the removals of nitrogen and phosphorus were reported [19], [22]–[24]. Among various NPs, CuNPs are one of the most important engineered nanoparticles which are used in a wide range of applications including fungicides, cosmetics, printers, and electronics [25]. However, the effect of CuNPs on sludge physical-chemical properties has never been reported.

The objectives of this study were to investigate the influences of CuNPs on the physical-chemical properties (such as EPS, surface charge, hydrophobicity, flocculating ability, settleability and dewaterability) of activated sludge. In addition, possible mechanisms were explored to explain the changes of sludge physical-chemical performances induced by different concentrations of CuNPs, and the possible influence of these property changes on biological phosphorus removal.

## Materials and Methods

### Ethic Statement

The original seed sludge used in the EBPR reactor was withdrawn from the secondary sedimentation tank of Qu Yang Municipal WWTP in Shanghai, China. All necessary permits for collection of the sludge from this system were granted by Qu Yang Municipal WWTP and Shanghai Municipal Construction Bureau. All field work has been conducted according to the relevant national guidelines.

### Copper Nanoparticles

Commercially produced CuNPs (99.9% purity, 20–40 nm) were purchased from Alfa Aesar. The X-ray diffraction (XRD) analysis of the CuNPs was conducted using a Rigaku D/Max-RB diffractometer equipped with a rotating anode and a Cu Kα radiation source, which investigated the presence of CuO and Cu_2_O coating on the Cu core, and the result is shown in Figure S1 in File S1. The stock suspension of CuNPs (200 mg/L) was prepared by dispersing 0.2 g of NPs in 1 L MilliQ-water, followed by 2 h of ultrasonication (25°C, 500 W, 20 kHz). The average diameter of NPs in the stock suspension was determined to be 128 nm by dynamic light scattering (DLS) analysis using a Malvern Autosizer 4700 (Malvern Instruments, UK).

### Relative Hydrophobicity and Surface Charge of Activated Sludge

The parent sequencing batch reactor was operated over 100 d with synthetic wastewater (see Supporting Information for details) and achieved stable biological phosphorus removal efficiency (>99%). The relative hydrophobicity of activated sludge was determined by microbial adhesion to hydrocarbons (MATH) assay, which was performed with n-hexadecane (Sigma, purity >99%) [26], and was calculated as the percentage of the cells adhering to hexadecane: (1−B/A)*100%. After batch experiments with CuNPs in the EBPR (see Supporting Information) were conducted, the sludge was washed twice and resuspended in phosphate buffer (0.2 M, pH 7.4). The initial absorbance of sludge at 600 nm was measured as A. Then 5 mL of this sludge suspension was mixed with 1.5 mL hexadecane and vortexed for 1 min. After 15 min incubation at 30°C, the aqueous phase was carefully separated from the above hydrocarbon phase, and the aqueous absorbance of 600 nm was measured as B.

The net surface charge (SC) of sludge flocs was measured by colloidal titration [5]. A 1 mL sludge sample, diluted to 100 mL, was blended with an excess amount of polybrene (5 mL, 0.001 M, Sigma), followed by back titration with polyvinyl sulphate (0.001 M, Sigma) to a colorimetric endpoint indicated by Toluidine Blue (change in color from blue to pink). The titration was terminated when electrical neutrality was reached, as indicated by the change of color from blue to pink. An equal volume of polybrene in distilled water was used as blank. The SC was expressed as milliequivalents per gram of mixed liquid suspended solids (MLSS) of negative colloidal charge [meq/g MLSS].

### Extraction of EPS and Excitation-Emission Matrix (EEM) Fluorescence Spectroscopy Analysis

A heat extraction methods was used to extract the EPS of activated sludge [27]. The concentrations of protein and carbohydrate in the EPS extraction were respectively measured by the modified Lowry method and phenol-sulphuric acid method [28]. At the end of the batch experiments, the EPS was extracted and measured using EEM fluorescence spectroscopy to characterize the structure of EPS with luminescence spectrometry (F-4600 FL Spectrophotometer, Hitachi, Japan) [29]. Particulates in samples were removed using a 0.45 μm polytetrafluoroethylene (PTFE) membrane prior to EEM tests. To obtain the fluorescence of EEM, The excitation and emission slits were set to a 5-nm band-pass and the scanning speed was 1200 nm/min. The excitation and emission wavelengths were incrementally increased from 200 to 450 nm and 200 to 550 nm at 5 nm and 0.5 nm steps, respectively.

### Flocculating Ability of Activated Sludge

The method of estimating the flocculating ability (FA) of sludge was modified according to the literature [5]. Briefly, the 80 mL sample of sludge with a MLSS concentration of 3 g/L was transferred into a beaker placed on an ice bath and sonicated at an output level of 50 W for 15 s. A 10 mL aliquot of the suspension was centrifuged at 1200 rpm for 2 min and the absorbance of the supernatant was measured at 650 nm (C). The rest of the sonicated suspension was stirred on a magnetic stirrer, which was kept at a constant speed, and was set at a low level to keep the sludge in suspension and to allow reflocculation at ambient temperature for 15 min, after which a 10 mL aliquot was analyzed in the same way as before (D). The FA of the sludge flocs was calculated as FA = (1−D/C)*100%.

### Specific Growth Rate

The specific growth rate (u) was measured according to the method in the literature [30]. It was assumed that all ammonia-nitrogen (NH_3_-N) was used for the growth (the nitrification was inhibited by thiourea) and it was the only possible source of nitrogen. Based on this assumption, the active biomass was calculated considering the molecular formula for biomass C_5_H_7_NO_2_, which means that 8 mg of biomass contains 1 mg of N [31]. Thus, the consumed NH_3_-N can be converted to the newly increased biomass. Batch experiments were conducted with exposures of 0, 5, 30 and 50 ppm of CuNPs. The initial concentrations of NH_3_-N and thiourea were set as 35 and 10 mg/L, respectively. The concentrations of NH_3_-N were measured every half hour and the consumed NH_3_-N in the aerobic stage was used to calculate the specific growth rate since the NH_3_-N in the anaerobic stage had almost no consumption. The average specific growth rate was calculated as: 
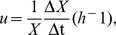
where X stands for the initial active biomass (mg), ΔX stands for the newly increased biomass (mg), and Δt stands for the corresponding time (h).

### Specific Oxygen Uptake Rate (SOUR)

The SOUR was measured following the modified method according to the literature [32]. In brief, 3000 mg/L of activated sludge amended with 0, 5, 30 and 50 ppm of CuNPs was fully aerated, and 100 mL of the fully aerated mixed liquor was added to the biological oxygen demand (BOD) bottle. The dissolved oxygen (DO) value in each bottle was measured at 1 min intervals until the DO was completely exhausted. The SOUR can be calculated by using the equation: 
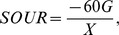
where G is the slope of the linear portion of the DO decline curve in mg/(L min), X is the biomass concentration in g/L, and the unit for the SOUR is mg O_2_/(g MLSS.h).

### Cell Membrane Integrity Assay

The cell membrane integrity of activated sludge was assayed by measuring the lactate dehydrogenase (LDH) release [33]. The LDH cytotoxicity detection kit (Roche Applied Science) was used to determine the LDH release according to the manufacturer's instruction. At the end of the experiments, the aliquots were centrifuged at 12000 *g* for 5 min and the supernatant was transferred to a 96-well plate, followed by adding 50 uL of substrate mixture (Roche Applied Science). Then 50 uL of stop solution (Roche Applied Science) was added to each well after incubation at room temperature for 30 min in dark, and the absorbance was recorded at 490 nm using a microplate reader (BioTek, USA).

### Analytical Methods

The analyses of soluble orthophosphorus (SOP), NH_3_-N, sludge volume index (SVI), MLSS, and mixed liquid volatile suspended solids (MLVSS) were conducted according to Standard Methods [34]. The capillary suction time (CST) was analyzed by CST instrument (model IFP-9050, Venture Innovations Ltd., USA) with Whatman No. 17 filter paper. The activated sludge amended with CuNPs was freeze dried and the surface morphology of sludge was imaged by the Environmental Scanning Electron Microscopy (ESEM, FEI QUANTA 250).

### Statistical Analysis

All tests were performed in triplicate and the results were expressed as mean ± standard deviation. An analysis of variance (ANOVA) was used to test the significance of results, and p<0.05 was considered to be statistically significant.

## Results and Discussion

### Effects of CuNPs on the EPS of Activated Sludge

The EPS carry negative charge at neutral pH value due to their negative charged functional groups. They can interact with cations in wastewater, forming the bridges, which can help to aggregate and stabilize the matrix of biopolymers and microbes, and therefore promoting the bioflocculation. EPS mainly include proteins, carbohydrates, humic acids and nucleic acids. As there were little amounts of humic acids and nucleic acids, the sum of protein and carbohydrate was considered as the total EPS in this study. It can be seen in Table 1 that the content of protein was almost kept constant under the exposure of different concentrations of CuNPs when compared to the control test (p>0.05, see Table S1 in File S1, statistical analysis). However, the amount of carbohydrate was enhanced with CuNPs, which resulted in the ratios of protein to carbohydrate declining from 5.2 (control) to 4.4 (30 ppm CuNPs), and then further to 3.8 (50 ppm CuNPs).

**Table 1:** Effects of CuNPs on the EPS Content of Activated Sludge.^a^

	Control	CuNPs (5 ppm)	CuNPs (30 ppm)	CuNPs (50 ppm)
Protein^b^	182.1±2.1	182.6±2.0	181.9±1.5	184.3±3.4
(% of the control)	(100)	(100.3)	(99.1)	(101.3)
Carbohydrate^b^	35.2±1.5	36.5±1.6	41.8±2.0	48.2±2.2
(% of the control)	(100)	(103.7)	**(118.8)**	**(136.9)**
Total EPS^b^	217.3±1.8	219.1±3.3	223.7±3.3	232.5±5.5
Protein/Carbohydrate	5.2	5.0	4.4	3.8

a The data reported are the averages and their standard deviations in triplicate tests.

b The units of protein, carbohydrate, and total EPS are mg COD/g MLSS.

**Figure 1:**
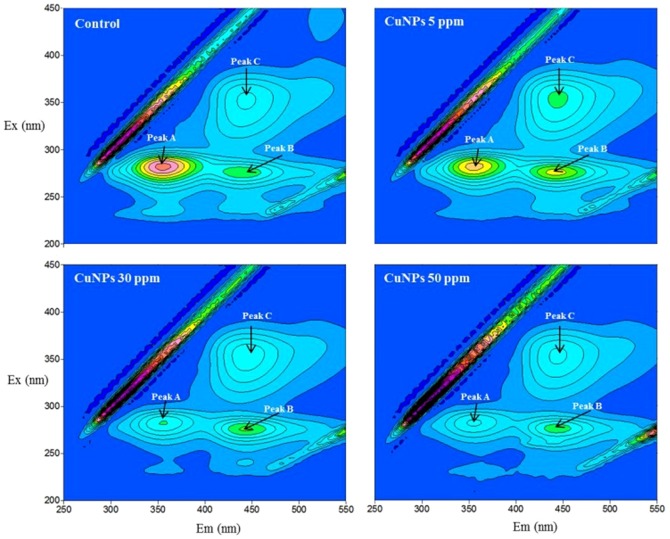
The EEM profiles of sludge EPS under the exposure of different CuNPs concentrations.

The data in Table 1 indicated that protein was the main component of EPS. It contains large quantities of aromatic structures, which have fluorescence characteristics. In this paper, the EEM was utilized to investigate the influence of CuNPs on the EPS structure. The main fluorescence peaks are shown in Figure 1, and the intensities of these peaks are illustrated in Table S2 in File S1. Peak A (Ex∼280 nm, Em∼355 nm) originated from the protein-like substances containing tryptophan, and peak B (Ex∼275 nm, Em∼440–445 nm) and peak C (Ex∼355 nm, Em∼445 nm) were related to humic acid-like organic compounds. The fluorescence intensity of peak A quenched to 82.6%, 53.0%, and 45.4% of the control under the exposure of 5, 30, and 50 ppm of CuNPs, respectively. While the intensities of Peak B and Peak C had no significant variation. It was reported that the fluorescence quenching was induced by the binding of metal ions, which changed the electronic polarization of both the metal and the binding site, and the binding capacity was indicated by quenching degree [35]. Therefore, CuNPs or their dissolved Cu^2+^ ions showed strong binding capacity on EPS. In addition, the fluorescence peaks of organic compounds under the exposure of metal ions were reported to have red or blue shifts because of their structure change [36], [37]. The data in Table S2 in File S1 showed that the fluorescence of peak A and C had no shift and peak B only had a slight emission wavelength red shift (from 440 nm to 445 nm) in the presence of CuNPs, indicating that CuNPs or the dissolved Cu^2+^ ions had no significant effect on the structure of EPS.

### Effects of CuNPs on the Surface Charge and Hydrophobicity of Activated Sludge

It was reported that the amount of carbohydrate in EPS was increased and the protein kept constant when sludge bulking occurred, resulting in the sludge surface charge increasing from −0.7 to −0.3 meq/g-VSS with the decreased ratio of protein to carbohydrate from 1.25 to 0.6 [38]. As shown in Table 2, similar result was observed in this study, which was that the ratio of carbohydrate to protein was decreased from 5.2 to 3.8, and the surface charge was increased from −0.69 to −0.38 meq/g MLSS as the concentration of CuNPs was increased from 0 ppm to 50 ppm. Besides, 5, 30 and 50 ppm of CuNPs could dissolve the Cu^2+^ cations of 0.13, 0.65 and 0.98 ppm in synthetic wastewater (see experiment of CuNPs dissolution in synthetic wastewater, Supporting Information), and the dissolved Cu^2+^ would bind on the sludge surface negative functional groups, making a contribution to the increased surface charge.

**Table 2:** Effects of CuNPs on Surface Charge and Relative Hydrophobicity of Activated Sludge.^a^

	Control	CuNPs (5 ppm)	CuNPs (30 ppm)	CuNPs (50 ppm)
Surface Charge (meq/g MLSS)	−0.69±0.021	−0.63±0.021	−0.52±0.02	−0.38±0.021
Relative Hydrophobicity (%)	81.7±2.1	79.5±1.5	70.8±2.0	61.0±2.1

a The data reported are the averages and their standard deviations in triplicate tests.

Sludge surface hydrophobicity plays an important role in the bioflocculation as the hydrophobic interactions are important for the attachment of bacteria to the surface [4]. Generally, since the hydrophobic molecules are non-polar, they less readily mix in water compared to the polar molecules, and should contribute to the biding together of sludge flocs [7]. Cell surface charge has been reported to be related with hydrophobicity of bacteria. Considering the surface charge is related to the ionizable groups present on sludge surfaces, it increases the polar interaction of EPS with water molecules, and therefore, cells with a largely negative surface charge will appear more hydrophobic [7]. Table 2 showed that the measured relative hydrophobicity tended to decrease from 81.7% (the control) to 61.2% (50 ppm of CuNPs), which was consistent with the increased surface charge observed in this investigation.

### Effects of CuNPs on the Flocculating Ability of Activated Sludge

**Figure 2:**
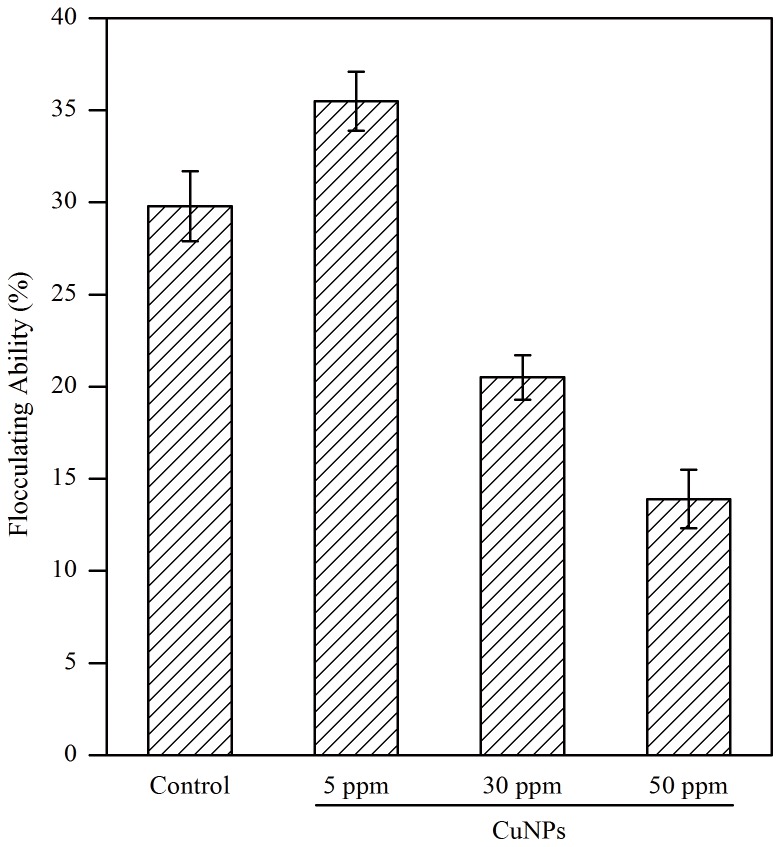
The effect of CuNPs on the flocculating ability of activated sludge. Error bars represent standard deviations of triplicate measurements.

The successful operation of biological wastewater treatment is governed by the ability of bacteria to induce floc formation to facilitate the separation of particles from the treated wastewater. As seen in Figure 2, different concentrations of CuNPs exerted different effects on bioflocculation. The exposure of 5 ppm of CuNPs showed a positive role in bioflocculation, while 30 and 50 ppm of CuNPs decreased the flocculating ability. It was documented that EPS composition, cell hydrophobicity and surface charge were related to the bioflocculation of sludge, and higher hydrophobicity and lower cell surface charge were in favor of bioflocculation [38], [39]. Under the exposure of 5 ppm of CuNPs, no significant variation occurred in the properties of EPS, surface charge and hydrophobicity. Additionally, the dissolved Cu^2+^ ions from CuNPs could function as the bridges between the negative charged functional groups on the surface of bacteria and help to aggregate the microbes, thus the promoted bioflocculation was observed at 5 ppm of CuNPs. However, these properties performed differently under the exposure to 30 and 50 ppm of CuNPs. The increased cell surface charge weakened the strength between EPS and cations, and the decreased hydrophobicity decreased the hydrophobic interactions between microbial surfaces, which would deteriorate the flocculating ability of activated sludge. Besides the CuNPs would induce the changes of these properties, it has been proven that the toxicity of NPs could change the cell membrane of bacteria, which would lead to the death of cell [40]. Thus, the exerted toxicity under the exposure of higher concentrations of CuNPs might be another reason for the declined flocculating ability, which will be investigated next.

**Figure 3:**
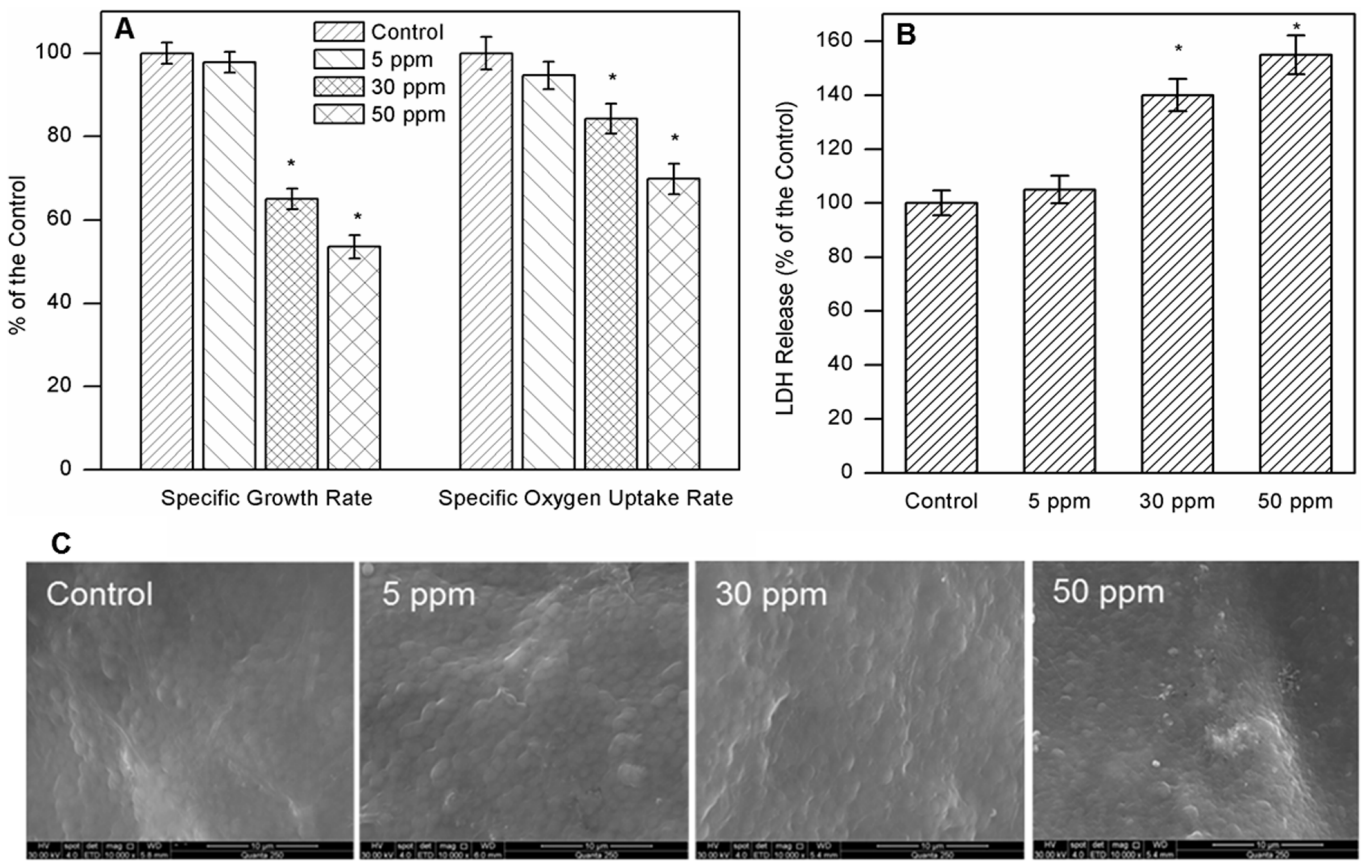
The toxic effect of CuNPs on activated sludge. The microbial activity was measured by the specific growth rate and specific oxygen uptake rate (A); the cell integrity was assessed by the LDH release (B) and the ESEM images (C). Asterisks indicate statistical differences (p<0.05) from the control, and error bars represent standard deviations of triplicate tests.

The possible toxicity of CuNPs to activated sludge process was examined by monitoring the changes of specific growth rate and specific oxygen uptake rate since these measurements permitted instantaneous responses to the injection of toxins [41], [42]. As shown in Figure 3A, 5 ppm of CuNPs had no significant adverse effects on the specific growth rate and specific oxygen uptake rate when compared with the control (see Table S3 in File S1, statistical analysis). However, the specific growth rate was decreased to 65.0% and 53.6% of the control, and the SOUR was declined to 84.3% and 69.8% of the control under the exposure of 30 and 50 ppm of CuNPs, respectively. Thus, higher concentrations of CuNPs showed inhibitory effects on the activity of activated sludge microorganisms, which was consistent with the phenomenon of detrimental bioflocculation. Usually, microbes exert the defensive response to alleviate the toxicity. EPS can serve as the protective layer of activated sludge, and some metal ions or metal nanoparticles have been reported to enhance the production of EPS to reduce their toxicity by immobilizing the metals [37], [43]. Therefore, the increased EPS content observed in this study might be stimulated by the toxicity of CuNPs.

**Figure 5:**
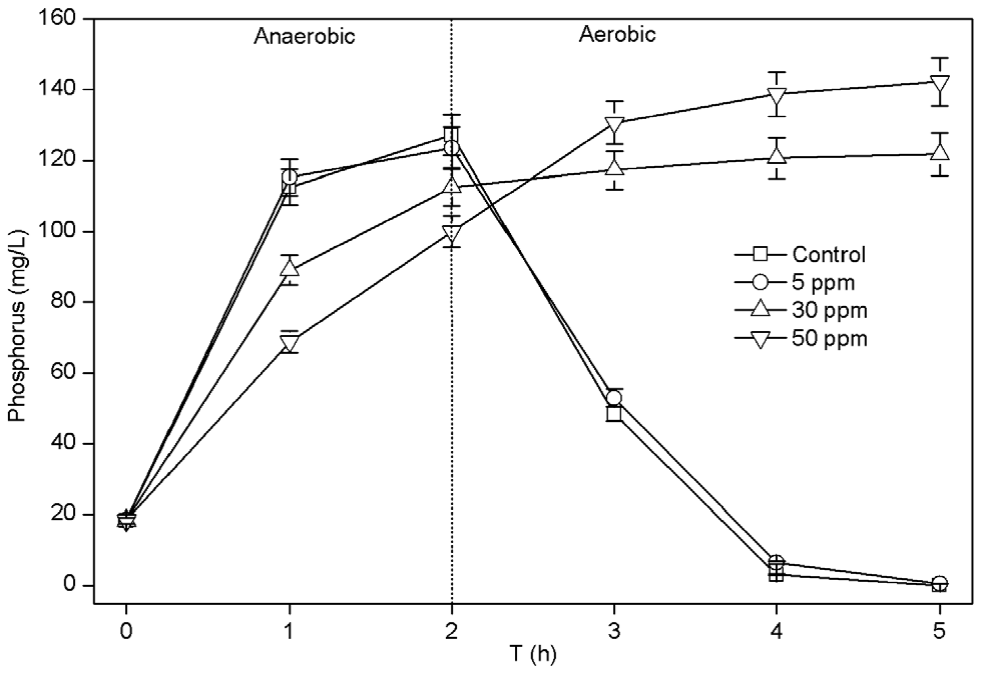
Effects of CuNPs on phosphorus anaerobic release and aerobic uptake during one cycle of EBPR process. Error bars represent the standard deviations of triplication tests.

The cell surface plays a vital role in the bioflocculation since it occurs between microbe surfaces. Further investigation on the integrity of cell membrane was conducted *via* LDH release and the surface morphology. Figure 3B showed that the LDH release (cell membrane damage marker) was enhanced with the increase of CuNPs, indicating that the cell leakage occurred (Table S4 in File S1, statistical analysis). The surface morphology images by ESEM analyses (Figure 3C) confirmed that higher concentrations of CuNPs (30 and 50 ppm) could damage cell membrane since cell dehydration and morphology changes were observed. Thus, the damaged cell membrane might contribute to the bioflocculation deterioration as well. Besides, the toxic CuNPs or dissolved Cu^2+^ might occupy the biding sites of Ca^2+^ or Mg^2+^ in the wastewater on the cell surface, resulting in the Ca^2+^/Mg^2+^-cation-induced bioflocculation weakening, which might be another reason for the deceased flocculating ability.

### Effects of CuNPs on the Settling and Dewatering Capacities of Activated Sludge

**Figure 4:**
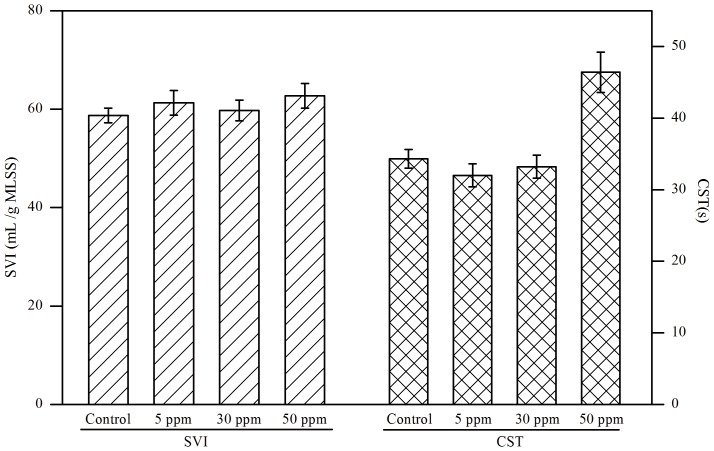
The effects of different concentrations of CuNPs on the settling and dewatering of activated sludge. Error bars represent standard deviations of triplicate tests.

In the literature different observations were made between bioflocculation and settleability/dewaterability of activated sludge. For example, some researchers reported that high flocculating ability was in favor of settling and dewatering capacities, while others found that there was no direct relationship between them for some activated sludges [4], [44]. In this study the data in Figure 4 indicated that the settleability of activated sludge was un-affected by CuNPs even at 50 ppm exposure when compared with the control (see Table S5 in File S1, statistical analysis). However, the dewatering capacity was decreased under 50 ppm of CuNPs exposure, and no significant variation occurred at the concentrations of 5 and 30 ppm (see Table S5 in File S1, statistical analysis). Thus, the exposure of CuNPs of these different concentrations had no effects on settlability, while the dewaterability was detrimented at high concentration exposure (50 ppm).

Since the physical-chemical properties changed, especially the declined flocculating ability, the activated sludge was apt to exist as dispersive small particles, providing a larger area to interact with CuNPs, which might enhance the toxicity of NPs to bacteria. Therefore, the exerted toxicity of CuNPs showed detriment to the flocculating ability of activated sludge, and the deteriorated bioflocculation might strengthen the toxicity of CuNPs in turn. Thus, the changes in the physical-chemical properties, especially the flocculating ability, can be considered as an indicator of the toxicity induced by the CuNPs.

Since the physical-chemical properties were deteriorated, such as the decrease of flocculating ability and the damage of cell membrane, the treatment efficiency of activated sludge, such as the phosphorus removal efficiency might be influenced. As seen in Figure 5, the presence of 5 ppm of CuNPs had no significant impact on phosphorus removal. However, 30 and 50 ppm of CuNPs had serious effects on both anaerobic and aerobic phosphorus transformations. The anaerobic phosphorus release, compared with the control (127.2 mg/L), was decreased respectively to 112.6 and 100.0 mg/L at 30 and 50 ppm of CuNPs, and there was no phosphorus uptake occurred in the aerobic stage. Thus, higher concentrations of CuNPs (30 and 50 ppm) had serious inhibition to phosphorus removal, which was consistent with the detrimental physical-chemical properties. Therefore, using the deteriorated physical-chemical property as an indicator of the toxicity of CuNPs to activated sludge was reasonable.

## Conclusions

The above studies indicated that most of the physical-chemical properties of activated sludge were unaffected by the exposure of lower CuNPs (5 ppm). However, these properties had different performances at higher CuNPs (30 and 50 ppm). It was observed that the hydrophobicity was decreased, the sludge surface charge and the EPS content were enhanced, and the flocculating ability was deteriorated. The worsened flocculating ability made the sludge flocs more prone to exist as dispersed form, which could further increase the toxicity of CuNPs. Further investigation indicated that the phosphorus removal efficiency was declined at higher CuNPs, which was in line with the deteriorated physical-chemical properties of activated sludge. Thus, the exposure of CuNPs to WWTPs resulted in the changes of physical-chemical properties of activated sludge, which provided an easy and simple method to assess the risk induced by nanoparticles.

## Supporting Information

File S1 **Combined file of supporting figures and tables.** Figure S1. The XRD pattern of the CuNPs used in this study. Table S1. The statistical analysis results of different concentrations of CuNPs affecting the content of the protein, carbohydrate and the total EPS (compared with the control). Table S2. Effects of CuNPs on the fluorescence spectral parameters of EPS. Table S3. The statistical analysis results of different concentrations of CuNPs affecting the specific growth rate and specific oxygen uptake rate (compared with the control). Table S4. The statistical analysis results of different concentrations of CuNPs affecting the LDH release (compared with the control). Table S5. The statistical analysis results of different concentrations of CuNPs affecting the settling (SVI) and dewatering (CST) (compared with the control).
(ZIP)
