# Supplementary material for: Influence of Copper Nanoparticles on the Physical-Chemical Properties of Activated Sludge
Source: PLoS One. 2014 Mar 24;9(3):e92871. doi: 10.1371/journal.pone.0092871 (PMC3963960; doi:10.1371/journal.pone.0092871)
Supplement: File S1 — Combined file of supporting figures and tables. Figure S1. The XRD pattern of the CuNPs used in this study. Table S1. The statistical analysis results of different concentrations of CuNPs affecting the content of the protein, carbohydrate and the total EPS (compared with the control). Table S2. Effects of CuNPs on the fluorescence spectral parameters of EPS. Table S3. The statistical analysis results of different concentrations of CuNPs affecting the specific growth rate and specific oxygen uptake rate (compared with the control). Table S4. The statistical analysis results of different concentrations of CuNPs affecting the LDH release (compared with the control). Table S5. The statistical analysis results of different concentrations of CuNPs affecting the settling (SVI) and dewatering (CST) (compared with the control). (ZIP) [file pone.0092871.s001.zip › Supporting Information/Supporting Information-revised.doc]

**Supporting Information**

**Influence of the Copper Nanoparticles on the Physical-Chemical Properties of Activated Sludge**

Hong Chen, Xiong Zheng, Yinguang Chen*, Mu Li, Kun Liu, Xiang Li

(*State key laboratory of pollution control and Resource reuse, School of Environmental Science and Engineering, Tongji University, 1239 Siping Road, Shanghai 200092, China*)

*Corresponding author

E-mail: [yinguangchen@tongji.edu.cn](mailto:yinguangchen@tongji.edu.cn)

Tel: 86-21-65981263

Fax: 86-21-65986313

**Materials and Methods**

**Set-up and Operation of Parent Sequencing Batch Reactor (SBR)**

The EBPR activated sludge was cultured with synthetic municipal wastewater (Supporting Information) in an anaerobic-aerobic SBR, which was covered by foil on the outside and had a working volume of 4 L with a cycle of 8 h consisting of 2 h anaerobic and 3 h aerobic periods, followed by 1 h settling, 10 min decanting and 110 min idle periods. The reactor was maintained at 21±1 ^0^C and constantly mixed with a magnetic stirrer except during the settling, decanting and idle periods. The influent pH was adjusted to 7.5 by adding 4 M NaOH or 4 M HCl. In the aerobic stage, air was provided by an aerator using an on/off control system and the dissolved oxygen (DO) concentration maintained at around 6 mg/L. After the settling period, 3 L of the supernatant was discharged, and replaced with 3 L fresh synthetic wastewater during the next initial 10 min of the anaerobic time. Sludge was wasted at the end of aerobic periods to keep the solids retention time (SRT) at approximately 10 d. The initial BOD concentration in the SBR was increased progressively over a 30 d period from around 80 to approximately 300 mg/L. The beginning ratio of BOD/SOP was 16, and it was increased to the final value of 20. After around 100 days’ acclimatization, the phosphorus anaerobic release and aerobic uptake as well as net removal in the SBR reached relatively stable. The mixed liquid suspended solids (MLSS) and mixed liquid volatile suspended solids (MLVSS) were 3200 ± 190 and 2380 ± 126 mg/L in the parent SBR.

**Synthetic Municipal Wastewater**

The synthetic wastewater included stock SOP solution named “P - water”, “concentration solution”, “trace-element solution” (adapted from Smolders et al.), a certain amount of carbon source and tap water. The P-water contained (g/L): 23.5 KH_2_PO_4_ and 17.6 K_2_HPO_4_·3H_2_O. The concentrated solution contained (g/L): 25.88 peptone, 4.24 yeast extract, 33.94 MgCl_2_·6H_2_O, 19.09 MgSO_4_·7H_2_O, 8.91 CaCl_2_·2H_2_O, 8.91 NH_4_Cl and 0.11 allylthiourea (nitrification inhibitor). The trace-element solution contained (g/L): 1.50 FeCl_3_·6H_2_O, 0.03 CuSO_4_·5H_2_O, 0.12 MnCl_2_·4H_2_O, 0.06 Na_2_MoO_4_·2H_2_O, 0.12 ZnSO_4_·7H_2_O, 0.15 CoCl_2_·6H_2_O, 0.18 KI, 0.15 H_3_BO_3_ and 10 ethylenediamine tetraacetic acid. One liter of synthetic wastewater contained 1.94 mL of P-water, 2.14 mL of concentrated solution, 0.27 mL of trace-element solution, and 0.267 mL of acetic acid. The synthetic wastewater final composition of the influent contained (mg/L): 55.38 peptone, 9.07 yeast extract, 72.63 MgCl_2_·6H_2_O, 40.85 MgSO_4_·7H_2_O, 19.07 CaCl_2_·2H_2_O, 19.07 NH_4_Cl, 0.24 allylthiourea, 0.41 FeCl_3_·6H_2_O, 0.01 CuSO_4_·5H_2_O, 0.03 MnCl_2_·4H_2_O, 0.02 Na_2_MoO_4_·2H_2_O, 0.03 ZnSO_4_·7H_2_O, 0.04 CoCl_2_·6H_2_O, 0.05 KI, 0.04 H_3_BO_3_, 2.70 ethylenediamine tetraacetic acid, 45.59 KH_2_PO_4_, 34.14 K_2_HPO_4_·3H_2_O and 280 acetic acid.

**Batch Experiments with CuNPs in EBPR**

The 1600 mL of mixture withdrawn from the parent SBR before the end of aerobic stage was centrifuged at 4000 rpm for 5 min, washed with 0.9% NaCl solution for 3 times, and resuspended in 400 mL of [distilled](app:ds:distilled) [water](app:ds:water) before being divided into 4 batch reactors which were covered by foil on the outside. Then 10, 60, 100 mL of CuNPs stock suspension (200 mg/L) were added respectively to reactors #1 - #3 to make then concentrations of 5, 30, and 50 ppm. Reactor #4, with no CuNPs addition, was served as the control. The 100 mL of resuspended sludge and 100 mL of synthetic wastewater (Supporting Information) were fed into each reactor. Distilled water was added to make the final volume of the mixture in each reactor to be 400 mL, resulting in the initial concentrations of BOD 300 mg/L and SOP 15 mg/L. The initial pH in each reactor was adjusted to 7.5 by adding 2 M NaOH or 2M HCl. After being bubbled with nitrogen gas for 10 min, all reactors were sealed and anaerobically stirred for 2 h, and aerobically stirred at DO of approximately 6 mg/L for 3 h.

**Experiment of CuNPs Dissolution in Synthetic Wastewater**

The released Cu^2+^ ion in synthetic wastewater due to the dissolution of CuNPs was determined according to the literature with some modification. Briefly, 5, 30 and 50 ppm of CuNPs in the synthetic wastewater were shaken for 5 h at 21 ± 1 ^o^C after 2h ultrasonication, and CuNPs were then removed by high speed centrifugation (14000 *g*) for 30 min. The supernatant was collected, filtered through 0.22 um mixed cellulose ester membrane, and determined by inductively coupled plasma optical emission spectrometry (ICP-OES, PerkinElmer Optima 2100 DV, USA) after acidified with 4% ultrahigh purity nitric acid.

**Reference**

1. Smolders GJF, Vandermeij J, Vanloosdrecht MCM, Heijnen JJ (1994) Model of the anaerobic metabolism of the biological phosphorus removal process - stoichiometry and pH influence. Biotechnol Bioeng 43: 461-470.

2. Kennedy AJ, Hull MS, Bednar AJ, Goss JD, Gunter JC, et al. (2010) Fractionating nanosilver: importance for determining toxicity to aquatic test organisms. Environ Sci Technol 44: 9571-9577.
