# Supplementary material for: Influence of Copper Nanoparticles on the Physical-Chemical Properties of Activated Sludge
Source: PLoS One. 2014 Mar 24;9(3):e92871. doi: 10.1371/journal.pone.0092871 (PMC3963960; doi:10.1371/journal.pone.0092871)
Supplement: File S1 — Combined file of supporting figures and tables. Figure S1. The XRD pattern of the CuNPs used in this study. Table S1. The statistical analysis results of different concentrations of CuNPs affecting the content of the protein, carbohydrate and the total EPS (compared with the control). Table S2. Effects of CuNPs on the fluorescence spectral parameters of EPS. Table S3. The statistical analysis results of different concentrations of CuNPs affecting the specific growth rate and specific oxygen uptake rate (compared with the control). Table S4. The statistical analysis results of different concentrations of CuNPs affecting the LDH release (compared with the control). Table S5. The statistical analysis results of different concentrations of CuNPs affecting the settling (SVI) and dewatering (CST) (compared with the control). (ZIP) [file pone.0092871.s001.zip › Supporting Information/Table S5.doc]

**Table S5 - The Statistical Analysis Results of Different Concentrations of CuNPs Affecting the Settling (SVI) and Dewatering (CST) (Compared with the Control).**

|  | | **CuNP (ppm)** | **F_observed_** | **F_significant_** | **P_(0.05)_** |
| --- | --- | --- | --- | --- | --- |
| SVI | 5 | | 10.67 | 7.71 | 0.19 |
|  | 30 | | 0.45 | 7.71 | 0.54 |
|  | 50 | | 5.54 | 7.71 | 0.08 |
| CST | 5 | | 3.76 | 7.71 | 0.12 |
|  | 30 | | 0.86 | 7.71 | 0.41 |
|  | 50 | | 58.28 | 7.71 | 7.13×10^-3^ |
